# Chemical Treatment of Bio-Derived Industrial Waste Filled Recycled Low-Density Polyethylene: A Comparative Evaluation

**DOI:** 10.3390/polym13162682

**Published:** 2021-08-11

**Authors:** Ishaq Sider, Mahmoud M. A. Nassar

**Affiliations:** 1 Department of Mechanical Engineering, Palestine Polytechnic University, Wadi Alhareya, Hebron P.O. Box 198, Palestine; 2 College of Applied Professions, Palestine Polytechnic University, Wadi Alhareya, Hebron P.O. Box 198, Palestine; mnassar@ppu.edu

**Keywords:** biocomposites, filler/polymer compatibility, chemical treatment, industrial waste, recycled polymer

## Abstract

The search for renewable alternatives for petroleum products that can be used in industrial applications is increasing. Each year, several tons of bio-derived industrial waste is produced and most of it is burned or placed in landfills. Olive pits (OP) have unique characteristics such as abundance, renewability, and biodegradability, which can be utilized to develop new types of biocomposites. One of the most promising uses of OP is that they can reinforce the mechanical properties of polymeric biocomposites. This study describes the preparation of recycled low-density polyethylene (rLDPE) that is filled with OP flour (10, 20, 30, and 40 wt.%) using a twin-screw extruder. The effects of the chemical treatment of the OP surface (sodium hydroxide (NaOH) and dimethyl sulfoxide (DMSO)) on the bio-filler/polymer compatibility along with the produced composite’s chemical, physical, mechanical, and thermal properties have been explored. Overall, the reinforced composites that were obtained with alkali-treated OP have better biocomposite properties. This indicates an improved compatibility between the bio-filler and matrix. The results are promising in terms of using OP flour in developing green composites.

## 1. Introduction

Natural fillers can be obtained from forestry and agricultural wastes, and this includes olive pomace, which is a by-product of the olive oil production industry. Considerable amounts of these wastes are produced, and they present an environmental hazard in olive oil-producing countries. Therefore, it is extremely important to safely handle such materials [1,2]. It is estimated that one ton of olives is responsible for producing 0.6 tons of olive mill solid residue [3,4]. Olive pits (OP) are residues that form part of the solid wastes produced by the olive oil manufacturing industry during the processing and extraction of olive oil from olives [5,6]. Some of the negative effects that result from the spread of olive solid waste in the fields are (i) inhibition of microbial activities, (ii) reduction in seed germination, (iii) and alteration of the soil characteristics in terms of the porosity and humus concentration. Accordingly, research for identifying new possible uses for the by-products of olive processing, particularly the solid ones, is crucial for the economy and environment [7,8,9]. The properties of olive stone flour, which include its abundance, biodegradability, ease of processing, low density, and low cost, make it a promising organic filler [10,11].

The properties of composites depend on a variety of factors such as the fiber–matrix adhesion, fiber length, fiber content (loading), fiber treatment, and fiber dispersion in the matrix [12,13,14,15]. When manufacturing natural-fiber-reinforced polymer composites, weak interfacial bonding occurs between the natural fibers and polymer matrices owing to the hydroxyl groups in natural fibers [16,17,18]. Extensive studies have been performed to understand the effect of chemical treatment on natural fibers. The hydrophilic nature of the natural fibers and the hydrophobic nature of the polymeric matrices leads to phase separation, thus resulting in weak bonding at the matrix–fiber interfaces of natural fiber composites. Chemical treatment of natural fibers decreases the inherent hydrophilicity of the fibers and improves the adhesion between the matrix and fibers [19,20].

The inconsistent performance of natural fillers compared to synthetic fillers is one of the main limitations for the commercialization of natural fillers. This inconsistency is due to the source of the natural fillers, leading to variations in the chemical composition of a plant, filler processing, and production methods [21,22]. However, the inclusion of natural fillers to strengthen polymers depends mainly on the properties of the fillers. In addition, the geometry, defects, inconsistency, crystallinity, and structure of the fillers are other factors influencing the behavior of the fillers. Hence, the morphology, mechanical properties, and chemical composition of the fillers can be significantly controlled and improved through biological, physical, and chemical treatments [23]. Among these, chemical treatments, including alkali treatment (mercerization), bleaching, acetylation, and benzoylation, are currently the most popular for natural fillers. The chemical treatment of fibers includes leaching out amorphous–nanocrystalline–biomass materials and removal of surface impurities and other substances. Therefore, the treated fillers reinforce the polymers by functioning as load-carrying elements owing to the improved filler–polymer compatibility, which provides strength and rigidity to the produced biocomposites [24,25]. Such treatments roughen the surface of the natural filler, and the removal of surface impurities promotes better filler–polymer interfacial compatibility and bonding, thus improving the overall performance of the produced biocomposites [26].

Thermoplastic polymer composites have been studied and researched extensively owing to their low cost and excellent mechanical properties. Research has particularly focused on the utilization of renewable resources that are being integrated into composite manufacturing owing to socioeconomic pressures for producing biodegradable materials and lowering costs [27,28,29]. The most common polyethylene types are low-density polyethylene (LDPE) and high-density polyethylene (HDPE). LDPE provides several advantages in the automotive industry such as fuel savings, chemical resistance, ease of processing, corrosion resistance, and electrical insulation, as reported in a previous study [30]. However, the use of recycled LDPE (rLDPE) as a polymer matrix for producing reinforced composites can be a serious environmental problem owing to the non-biodegradable properties of LDPE. Among the few studies on rLDPE-based bio-composites, a noteworthy one conducted by Youssef et al. [28] demonstrated that the tensile strength of the composites increases as the fiber percentage increases up to 10% and then slightly decreases. Nevertheless, as the fiber content increases, several issues related to the microstructure are observed, which deteriorate the mechanical properties [31]. Owing to the hydrophilic nature of rice husk, the strength of rice husk/rLDPE composites decreases with the increasing natural filler content [32]. However, the incorporation of up to 6% nanosilica and 4% nanoclay was determined to be optimal. Excessive amounts of nanoparticles can agglomerate, resulting in gaps and cracks in the prepared eco-composites [33]. Meanwhile, incorporating rLDPE with up to 40 wt.% of cocoa waste degrades the strength and elongation, and the material rigidity increases [34].

Hence, based on the existing studies [3,35], we concluded that LDPE and OP waste are abundant but not widely used in producing materials, particularly biocomposites. Therefore, this study evaluated the various properties of natural-filler-based polymer biocomposites fabricated from the residue of OP and rLDPE in the form of a powder. The effect of using chemically treated OP as the raw material on the performance of the produced biocomposite was investigated. The physical, chemical, thermal, and mechanical properties of the developed biocomposites were thoroughly analyzed in this investigation.

## 2. Materials and Methods

### 2.1. Materials

**Figure 1:**
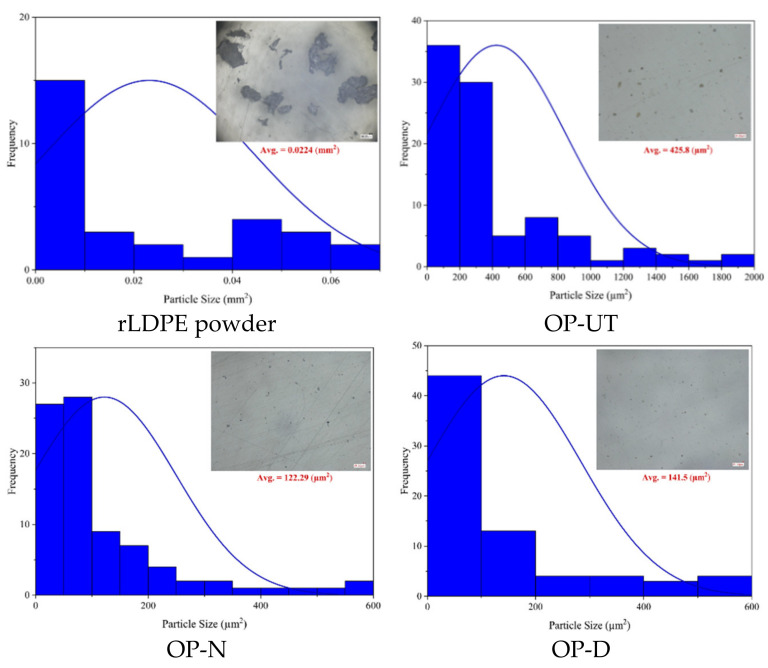
Particle size measurements of the used materials.

The rLDPE powder was acquired from Suzhou Poks Machinery Co., Ltd. (Suzhou, China). The OP residues were provided by a local industry in Palestine. First, the OP residues were powdered by using a flour mill grinding machine, followed by powder sieving with a mesh size of 100. The raw powder was used as a filler for the rLDPE matrix without any modification, and it was denoted by OP-UT. In addition, two more sets of samples were obtained: (1) by treating the powder using NaOH as described in [36], which was denoted by OP-N; (2) by treating with distilled water/ dimethyl sulfoxide (DMSO) (50/50 wt.%) for 2 h at 100 °C, which was denoted by OP-D. The properties of the rLDPE used in this study were first measured and reported for comparison purposes, as shown in Table 1. The optical microscopy (VHX-5000 series, Osaka, Japan) images of the rLDPE and used fillers were processed with the ImageJ software program to obtain the particle size distribution, as shown in Figure 1.

### 2.2. Composite Fabrication

Melt mixing followed by compression molding was used to fabricate the composite panels. The process started by preparing the composite ingredients based on the weight percentage according to a mold size of 230 × 230 × 2 mm. The mixing process was conducted using a twin-screw extruder (Tengda TSH-35P, Nanjing, China) with 10 consecutive heating zones, at a temperature range between 200 and 220 °C and with screws rotating at a speed at 200 rpm. Then, the mold in the compression molding machine (Carvar, Wabash, IN, USA) was filled with the mixture, and it was compressed between two heated plates at 190 °C under a pressure of 40 MPa. The compression was maintained for 15 min before the heating elements were switched off to let the panel cool down. The produced panels were cooled down and cured by applying tap water on the outer area of the heating plates of the hydraulic press machine for 2 min.

### 2.3. Characterization

#### 2.3.1. Chemical Characterization

A Fourier transform infrared (FTIR) spectrometer (Agilent Cary 630, Santa Clara, CA, USA) was used to analyze the chemical changes of the functional groups of the developed biocomposites. The samples were scanned over a range of 400 to 4000 cm^−1^ with a total of 64 scans at a resolution of 4 cm^−1^ at an ambient temperature.

#### 2.3.2. Physical Properties

The characterization was performed following the standards and procedures explained in an earlier study [17]. For the physical characterization, the density of the produced samples was measured with a densitometer (MZ-A300, Shenzhen Qun Long Instrument Equipment, Shenzhen, China). A 2 g specimen sample was placed in distilled water and the volumetric change in the water was measured at room temperature. The average volume of the five samples was measured and recorded.

To assess the water absorption of the developed biocomposites, procedures adopted from the ASTM D570 standard were followed. Five dried samples were soaked in distilled water at room temperature for 24 h. Then, the percentage of the water content that was absorbed by the biocomposite specimen was calculated by measuring the difference in the sample weights, before and after soaking it in water.

To evaluate the crystallinity of the untreated and treated fillers, the biocomposite sheets were examined at an ambient temperature through step scanning with an X-ray diffractometer (Rigaku Corporation, Tokyo, Japan). The measurements were carried out at 40 kV and 20 mA, with a detector mounted on a goniometer scanning scale from 10 to 60°, at a scanning speed of 5° min^−1^ by applying monochromatic CuKα radiation (λ = 1.5406 nm). The crystallinity degree was then measured as explained in an earlier study [17].

In addition, the melt flow index (MFI) (DRK208B Plastic Melt Flow Index tester, Qingdao, China) values of the developed biocomposites and pure polymers were measured at 190 °C with a standard weight of 2.16 kg, according to the ASTM D1238 standard [37]. For the latter, the MFI was determined using the average values of three samples.

#### 2.3.3. Mechanical Properties 

As for the mechanical characterization, the tensile properties were determined according to the procedures described in the ASTM D638 standard [17]. Tension tests were conducted using a universal testing machine (Tinius Olsen 10 kN, Redhill, UK) at a crosshead speed of 5 mm/min. Specimens were fixed vertically between the grips of the testing machine, which were tightened evenly and firmly to prevent any slippage, and the gauge length was kept 30 mm. The tests were conducted at room temperature. Five specimens (replications) were tested for each type of the developed biocomposites, and the average tensile properties were reported.

#### 2.3.4. Thermal Properties

For evaluating the thermal behavior, thermogravimetric analysis (TGA) and derivative thermogravimetric (DTG) plots were analyzed by using a thermogravimetric analyzer (TGA Q 500 TA Instrument, New Castle, DE, USA). The samples were deposited in an aluminum pan and heated in the range of 20–600 °C at a heating rate of 10 °C/min under an inert atmosphere. Then, the plots were analyzed using the TA Universal Analysis software.

## 3. Results and Discussion

### 3.1. Chemical Characteristics

**Figure 2:**
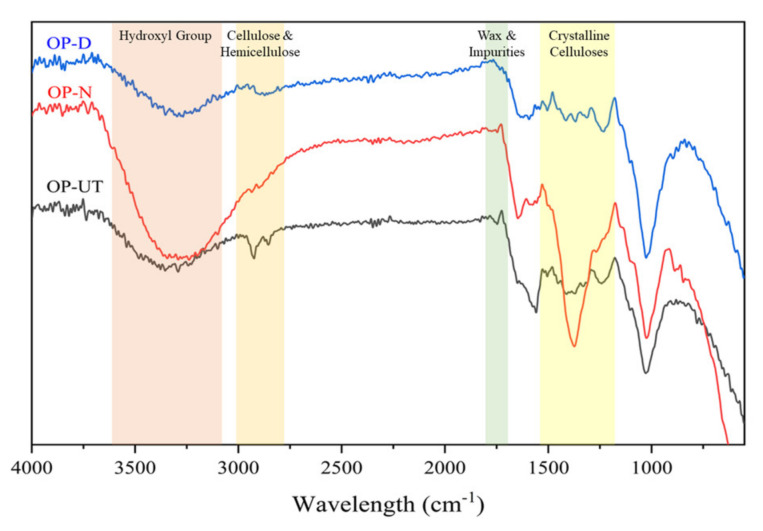
FTIR spectra of the raw filler (OP-UT) and treated filler (OP-N and OP-D).

After performing FTIR spectroscopy, as shown in Figure 2, it was observed that the major internal chemical composition of the filler was not altered because the treatment preserved the filler structural integrity. The FTIR spectra for all the experiments were almost consistent, which confirms that no additional absorption bands were applied to the filler. The main changes were detected by the disappearance of the peak around 1746 cm^−1^, after treatment with NaOH and water/DMSO, which is related to the wax and impurities on the filler surface. The FTIR spectra for the final yield of the treatment methods are presented in Figure 2. The band at 1378 cm^−1^ (Figure 2) showed significant differences in absorbance capabilities. This is the band that was assigned to both the crystalline celluloses (Cel I and Cel II), and after the treatment, this band became more intense when treated with NaOH compared to the untreated and water/DMSO treated fillers [38]. The differences between the NaOH and water/DMSO treatment can be depicted as the higher intensity of the cellulose backbone (1025 cm^−1^) and OH group (around 3316 cm^−1^), as well as the lower intensity of the hemicellulose peak (2861 cm^−1^) in the case of NaOH. Moreover, the OH group band increased significantly when it was treated in comparison to the untreated OP. This occurred because the treatment steps removed the amorphous biomass and increased the cellulosic content exposure of the filler, which favored access to the OH groups.

**Figure 3:**
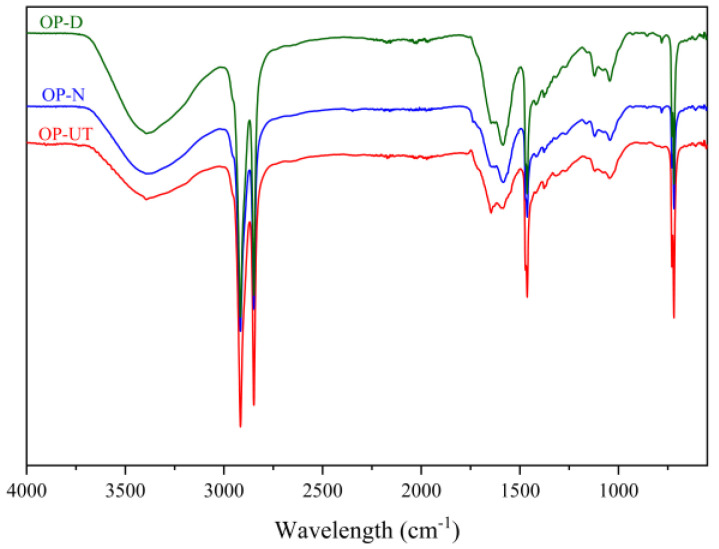
FTIR spectra of the developed biocomposites.

Figure 3 presents the spectra of the developed biocomposites in comparison to the rLDPE. The FTIR spectrum of the developed biocomposites does not show any changes in the peaks, but the peaks are clear and intense in the case where the treated OP was used as a filler. The absorption peaks that appeared for both composites at 1053 and 1368 cm^−1^ are assigned to the CH_3_ rocking vibration, and the peak at 3375 cm^−1^ is assigned to the symmetric bending vibration mode of the CH_3_ group. These absorption bands are normally associated with the presence of cellulose, hemicellulose, and lignin. However, the intensity of these peaks became more pronounced in the biocomposite filled with treated OP in comparison to the biocomposite treated with the treatment steps that exposed the content rich in cellulose [39].

### 3.2. Tensile Properties

**Figure 4:**
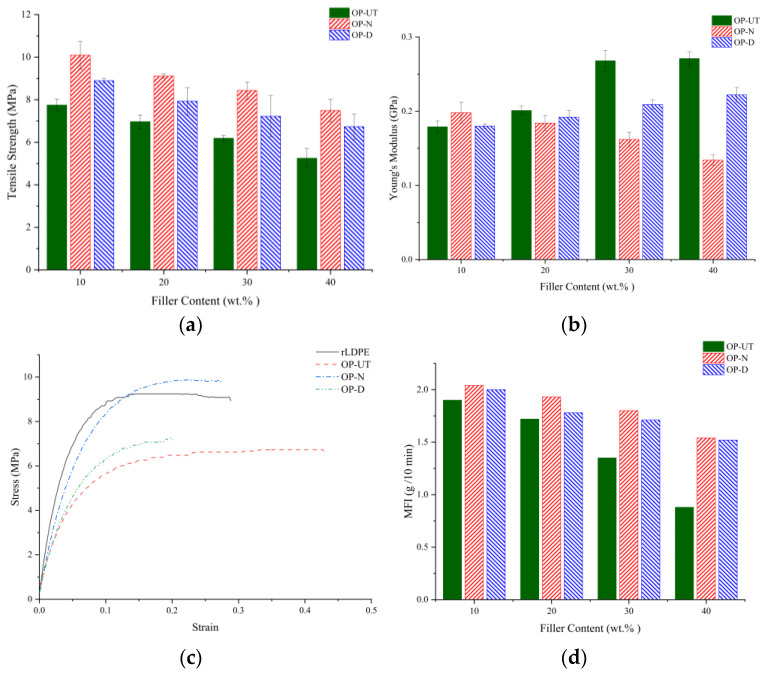
Tensile properties of the developed biocomposites: (**a**) tensile strength, (**b**) Young’s modulus, (**c**) stress–strain curve of developed biocomposite at 20% OP filler content, and (**d**) MFI values of the developed biocomposites.

Several factors, such as adhesion at the interface between the polymer and filler and the mechanical properties of the matrix and filler, can have a substantial effect on the strength and performance of the biocomposites. For particulate composites, the effectiveness of the load transfer between the matrix and filler is dependent on many factors such as the particle size, dispersion/distribution state, surface area, and particle filling. The improved surface topography of the chemically functionalized filler is expected to improve the filler/polymer interfacial adhesion to the matrix. The tensile strength, Young’s modulus, and MFI of the rLDPE-based biocomposites that use these filler types are presented in Figure 4a–c. A good adhesion between the reinforcement filler and rLDPE interphase results in increased tensile strength of the reinforced biocomposites, as shown in Figure 4a. Overall, it can be shown that the biocomposites based on treated fillers had an improved tensile strength compared to the neat polymer and untreated biocomposites. The findings show that the biocomposites based on NaOH-treated filler significantly improve the compatibility between the filler and the matrix, thus resulting in better mechanical properties. This is attributed to the increase in the interfacial adhesion between the filler and the rLDPE matrix.

The Young’s moduli of the different biocomposites and stress–strain diagram of the biocomposite with a 20% OP loading are shown in Figure 4b,c, respectively. However, the tensile modulus of the biocomposites based on untreated and treated filler behaved differently. Generally, an improvement in the tensile modulus was observed upon the addition of the filler, and a significant improvement was noticed by using the untreated filler in comparison to the neat polymer, especially for content that is more than 30 wt.%. However, the modulus decreases with the increase in the treated filler content, and there was a significant change with the filler that was treated with NaOH. It is interesting to note that the MFI is inversely proportional to the filler content, and it decreases in comparison to the neat rLDPE, as shown in Figure 4c. This implies that rLDPE with the OP filler exhibits better wetting behavior with higher MFI. Thus, MFI might play a dominant role in increasing the tensile strength of the biocomposites because the mechanical properties of the polymers or their composites are inversely proportional to the MFI of the matrix polymer.

Table 2 compares the tensile properties of the developed rLDPE biocomposites with 20 wt.% OP with those of rLDPE composites containing other types of natural fillers. The data presented in Table 2 clearly indicate that the newly developed biocomposites show improved tensile properties.

### 3.3. Physical Properties

#### 3.3.1. Density

Density is a critical property of the biocomposites; it determines their applicability in many industrial sectors as an alternative to neat polymer or synthetic-filler-reinforced polymers. In Table 3, a reduction is observed in the biocomposite density due to the presence of the filler in comparison to the neat rLDPE, and it is inversely proportional to the filler content. The untreated fillers with rLDPE show the lowest density followed by the treated fillers. The treated fillers result in a slightly higher density of the corresponding biocomposites due to the chemical treatment effect and filler/polymer adhesion, which eliminate microvoids. Overall, the newly developed biocomposites in all the cases display similar density values, which are deemed suitable for a variety of applications, especially in cases where lightweight structures are desired.

#### 3.3.2. Water Absorption

**Figure 5:**
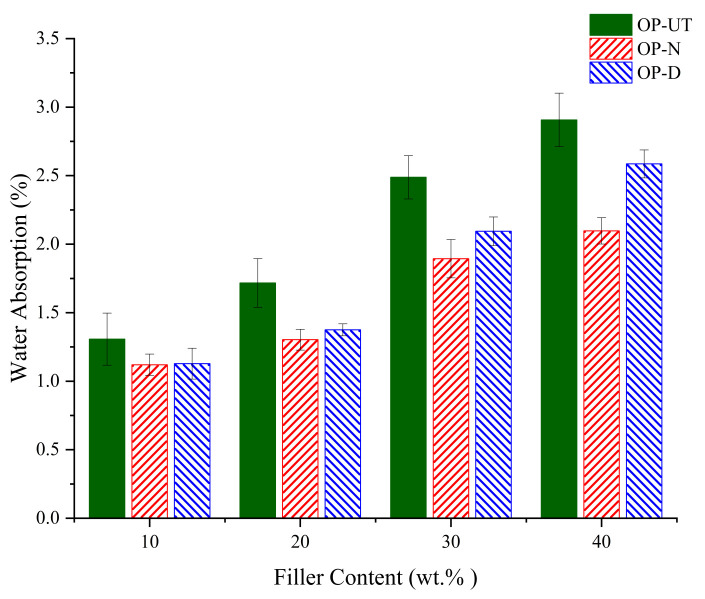
Water absorption of developed biocomposites.

Figure 5 presents the water absorption of the developed biocomposites when using the untreated and treated fillers. The water absorption characteristics are responsible for the filler characteristics. Generally, the use of the treated filler increases the water repellent properties of the biocomposites, which may be an indicator of good filler/polymer interfacial adhesion. A reduction in the water absorption that was observed in the treated filler indicates an improvement in the interfacial bonding between the treated filler and polymer matrix. This improvement was determined to be better in the case of the NaOH filler. This indicates that the NaOH treatment can better enhance the filler/polymer interfacial surfaces compared to the water/DMSO treatment. On the other hand, it was determined that the water absorption increases as the filler content increases. This is expected because the filler can absorb water due to its surface properties. Thus, it can be concluded that the treatment method is an appropriate method to decrease the absorption of water and improve the durability and stability of the developed biocomposites.

#### 3.3.3. X-ray Diffraction

**Figure 6:**
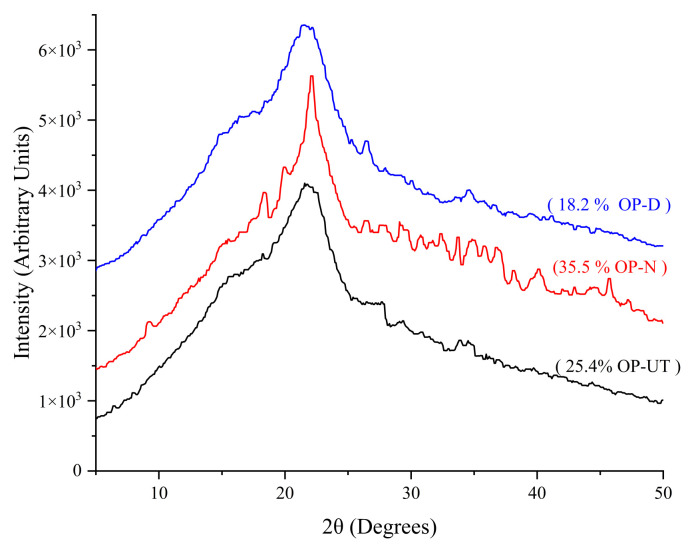
XRD patterns of the untreated and treated fillers.

The X-ray diffraction (XRD) patterns, presented in Figure 6, reflect the influence of the chemical treatment on the crystallinity degree of the fillers. The crystalline plane (002) corresponds to the intense broad peak at 2θ = 22°, which is attributed to cellulose and hemicellulose dehydrates. In general, the broad peak, as shown for all the samples, represents poor crystallinity and the amorphous nature of the biomass content. It was determined that the crystallinity degree of the NaOH-treated OP increased to 35.5%, while that of the untreated OP is 25.4%. Meanwhile, the water/DMSO treatment reduces the crystallinity of the OP to 18.2%. Therefore, it is expected that the water/DMSO treatment may have altered the structure of the OP, removing a substantial amount of biomass and decreasing the degree of crystallinity, as confirmed by XRD. The enhancement in crystallinity was observed in the NaOH-treated filler. This suggests that incorporating NaOH can partially remove the amorphous biomass without affecting the crystalline biomass, particularly for cellulose. Therefore, it is expected that the properties of biocomposites depend on the filler surface properties, filler/polymer adhesion, and filler integrity after the treatment.

**Figure 7:**
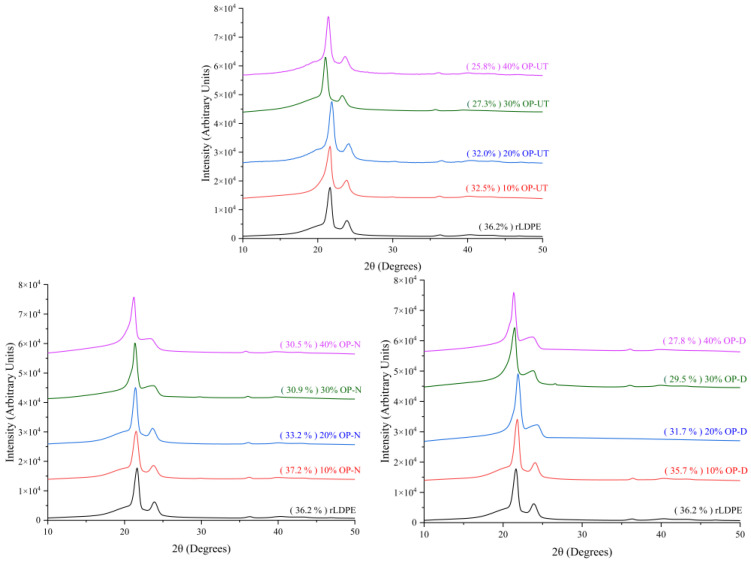
XRD patterns of the developed biocomposites in comparison to neat rLDPE.

Figure 7 exhibits the XRD pattern of the developed biocomposites in comparison to the neat polymer. The XRD pattern of the rLDPE and developed biocomposites show peak positions at 22° and 24°. The OP was embedded into rLDPE matrices, and no major effects appeared due to the filler addition. Hence, only the rLDPE characteristic peaks were observed. In general, it was observed that the filler content is inversely proportional to crystallinity degree in all the cases due to the polymer structure discontinuity because the filler is in particulate form. A slight increase was detected for the treated filler in comparison to the untreated filler, which indicates improved interfacial bonding of the filler/polymer surfaces. Improved adhesion was observed in the NaOH-treated filler, which agrees with the findings from the mechanical property tests.

### 3.4. Thermal Properties

**Figure 8:**
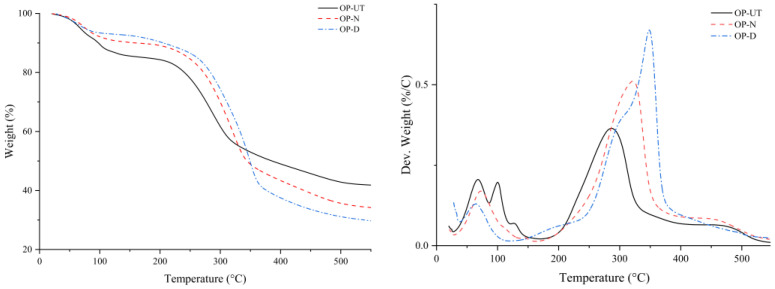
TGA/DTG curves of raw filler (OP-UT) and treated fillers (OP-N and OP-D).

**Figure 9:**
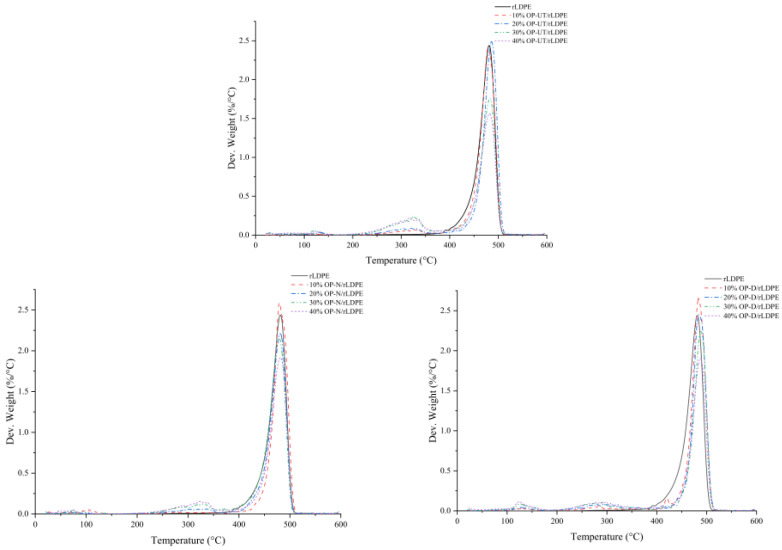
DTG curves of the developed biocomposites in comparison to rLDPE.

TGA/DTG was used to investigate the thermal behavior of OP-UT, OP-N, and OP-D under an inert atmosphere, as shown in Figure 8. Cellulose is the main component of OP. The decomposition of cellulose in an inert atmosphere is normally an endothermic process because cellulose is resistant to thermal degradation due to its crystalline nature. As seen in the TGA of the samples, the first decomposition zone (under 140 °C) displays a mass loss that is associated with the removal of moisture. The TGA curves, depicted in Figure 9, reveal an evident change in the thermal degradation process of the treated OP due to the removal of the wax and impurities along with the partial removal of the amorphous content. By increasing the temperature, as shown in the DTG, OP-UT and OP-N fillers undergo two subsequent degradation steps; the first zone is in the range of 200–330 °C and 200–380 °C for OP-UT and OP-N, respectively. This includes a major mass loss, which indicates an overlapping simultaneous degradation of holocellulose (cellulose and hemicellulose), whereas the second zone in the range between 430–490 °C is attributed to lignin degradation. It is noteworthy that lignin degradation proceeds gradually over a wider temperature range than cellulose and hemicellulose. However, for the OP-D, three degradation zones were observed in the ranges of 150–240 °C, 250–310 °C, and 310–390 °C. These are attributed to the degradation of hemicellulose; simultaneous degradation of hemicellulose, cellulose, and lignin; and simultaneous degradation of cellulose and lignin, respectively. Overall, the enhanced thermal characteristics of the treated filler confirm its potential use as a stable filler for the advanced synthesis of bio-composites even at high temperatures.

The weight loss and weight loss derivative (TGA/DTG) effects of the developed biocomposites at different filler weight percentages as opposed to the neat rLDPE can be observed in Figure 9. A slight weight loss between 100 and 115 °C that is also proportional to the filler percentage was observed. This could be attributed to water evaporation, which is indicated by the internal moisture in the filler. Next, degradation occurs in two stages; however, for the polymer, it occurs only in one zone between 400–500 °C. For the biocomposite degradation, the first stage is between 240 and 360 °C, which is related to the thermal degradation of hemicellulose and lignin in the filler. This zone can also be clearly observed in the case of rLDPE filled OP-UT, which denotes a higher impurities and amorphous content of the filler as it is degraded at a lower temperature in comparison to the crystalline content. The second stage suggests that the decomposition of the rLDPE matrix with the filler residue starts at 430 °C. Above 500 °C, the filler and polymer are completely decomposed with only residues of the biocomposite, which are related to the filler ash. However, the temperature at the highest decomposition percentage increases with an increase in the filler content. In contrast, the use of OP-D slightly increases the thermal stability of the biocomposites as shown in Figure 9. Hence, no significant shift/change is observed in the major degradation peak for the biocomposites based on treated filler.

## 4. Conclusions

In this study, two methods for the natural filler treatment of rLDPE-based biocomposites were developed, tested, and analyzed. The first scheme involves a commercial chemical treatment of the OP. The second scheme used water/DMSO for the surface filler treatment. To the best of our knowledge, this investigation implemented these methods for the first time with biocomposites. The results demonstrated that a good filler/polymer compatibility can be achieved by using these treatment methods. The NaOH treatment showed superior properties in comparison to the water/DMSO treatment, and this was supported by the findings from the mechanical and physical properties.

Further studies and statistical analyses are essential to explore the applications of these treatment methods and recycled OP in different types of polymers (virgin and recycled) for developing new classes of biocomposites. Additional coupling agents and compatibilizers should be identified and used in a controlled manner for producing high-performance biocomposites.

## Figures and Tables

**Table 1:** Properties of the rLDPE.

Property and Unit	Value
Tensile Strength (MPa)	8.78 ± 0.19
Young’s Modulus (GPa)	0.33 ± 0.02
Water Absorption (%)	0.04 ± 0.008
Density (g/cm^3^)	0.902 ± 0.012
MFI (g/10 min)	2.36 ± 0.25

**Table 2:** Tensile properties of natural-filler-reinforced rLDPE composites reported in the literature [25].

Filler Type		Filler Content (wt.%)	Tensile Strength (MPa)	Young’s Modulus (GPa)
Current Study	OP-UT	20	6.97	0.2
	OP-N	20	9.11	0.18
	OP-D	20	7.93	0.19
Corn husk fibers		5	24.7	0.33
Rice husk		5	9.3	0.55
Cocoa		10	6.9	0.16
Uncarbonized bagasse particles		20	9.2	0.07
Carbonized bagasse particles		30	11.5	0.09
Rice husk/Nanoclay		35	8	1
Rice husk/Nanosilica		35	14.5	0.8

**Table 3:** Density of developed biocomposites.

Filler Content (wt.%)	OP-UT	OP-N	OP-D
10	0.951	0.97	0.988
20	0.931	0.988	0.979
30	0.923	0.94	0.959
40	0.909	0.92	0.928
